# Effects of a Single Rapid Infusion System on Platelet Function in Stored Whole Blood: An Ex Vivo Study

**DOI:** 10.7759/cureus.16518

**Published:** 2021-07-20

**Authors:** Joshua W Sappenfield, Jeffrey D White, J. Peter R Pelletier, Tyler J Loftus, Faisal Mukhtar, Terrie Vasilopoulos, Shahrukh Bengali, Nikolaus Gravenstein, Ilan Keidan

**Affiliations:** 1 Anesthesiology, University of Florida College of Medicine, Gainesville, USA; 2 Pathology, University of Florida College of Medicine, Gainesville, USA; 3 Surgery, University of Florida College of Medicine, Gainesville, USA; 4 Anesthesiology/Orthopedics and Rehabilitation, University of Florida College of Medicine, Gainesville, USA; 5 Anesthesiology, University of Texas Southwestern Medical Center, Dallas, USA

**Keywords:** adenosine diphosphate, blood platelets, platelet count, platelet transfusion, thromboelastography

## Abstract

Introduction

Rapid infusion systems (RIS) are used to warm and rapidly infuse crystalloids and blood products. Current guidelines do not approve of platelet transfusion through a RIS, but data supporting these guidelines are scarce. Our hypothesis was that an infusion of whole blood through a RIS would degrade platelet quantity, impede viscoelastic clot strength, and inhibit platelet aggregation response to adenosine diphosphate pathway (ADP) activation.

Methods

Ten iterations of a simulated scenario of transfusing whole blood via a single brand and make of RIS (Belmont Fluid Management System 2000, Belmont Medical Technologies, Billerica, MA) were performed. Units of whole blood, which were two to nine days old, were leukoreduced prestorage. Blood was used to prime the RIS and then warmed and infused at 100 mL/min into a reservoir. Blood samples were collected before and immediately after infusion. Samples were tested for platelet count, size, and viscoelastic clot strength using thromboelastographic and aggregation assays.

Results

The study sample (n = 10) included platelets with an average age of 5.3 days. The infusion through the RIS had a detrimental effect on all the maximal amplitudes (MA) of viscoelastic testing: MA ADP (mean difference = −18.7 mm; 95% CI: −24.1 to −13.3, P = 0.004), MA rapid thromboelastography (MA rTEG) (mean difference = −6.0; 95% CI: −10.0 to −2.0, P = 0.008), MA TEG (mean difference = −7.1; 95% CI: −10.9 to −3.4, P = 0.004), mean platelet volume (MPV) (mean difference = −0.3; 95% CI: −0.6 to −0.1, P = 0.02), and platelet count (mean difference = −68.3 × 10^3^/µL; 95% CI: −86.9 to −49.7, P = 0.004).

Conclusions

Platelet quantity, viscoelastic clot strength, and platelet aggregation response to ADP each decline after infusion through a RIS. Further studies regarding microaggregates and platelet activation are required.

## Introduction

Exsanguinating hemorrhage is the second most common cause of death after traumatic injury and the most common cause of death within the first 48 hours following trauma in patients arriving alive to the hospital [1]. Along with operative and source control of the bleeding, protocol-driven massive blood product transfusion is associated with decreased mortality, shorter hospital and intensive care unit length of stay, fewer ventilator days, and decreased patient care costs [2-4]. Each institution differs with respect to the components of their massive transfusion protocol; however, when massive transfusion is administered in the setting of trauma, a ratio of packed red blood cells (PRBCs), plasma, and platelets is used, balancing clinical logistics and clotting substrate.

Massive transfusion is facilitated by rapid infusion systems (RIS), which can simultaneously warm and rapidly infuse blood products. Alternatively, platelets can be administered through a separate line from the RIS. Giving platelets via a warming RIS as part of a large volume resuscitation was described as early as 1991 [5]. Whole blood transfusions are replacing component blood therapy in some trauma centers during acute resuscitation, and in such instances, the use of a RIS for massive transfusion inexorably involves the passage of platelets through the RIS. However, manufacturer recommendations and current guidelines from the American Association of Blood Banks advocate avoiding platelet transfusion via RIS [6,7]. A suggested reason is that platelets adhere to the insides and reduce the number that makes it to the patient [8]. Data regarding the impact of warming and rapid infusion of platelets through a roller pump device such as the Fluid Management System 2000 are scarce. To address this knowledge gap, we constructed a simulated rapid trauma transfusion scenario to assess the effect of infusion via RIS on the platelet quantity, viscoelastic clot strength, and platelet aggregation response to adenosine diphosphate pathway (ADP) activation. Our hypothesis was that infusion through a RIS would have a negative effect on platelets as assessed by platelet quantity, viscoelastic clot strength, and platelet aggregation response to ADP activation.

Findings from this study have been presented as an abstract at the 2019 International Anesthesia Research Society Annual Meeting, 2019 Society of Critical Care Anesthesiologists Annual Meeting, and 2019 Association of University Anesthesiologists Annual Meeting.

## Materials and methods

This study was approved by the University of Florida Institutional Review Board (IRB201602434). A total of 10 trials were chosen a priori. The Fluid Management System 2000 (Belmont Medical Technologies, Billerica, MA) reservoir was filled with one unit of whole blood (two to nine days old), which was then used to prime the RIS circuit. The blood was prestorage leukoreduced (not irradiated), and the initial collecting anticoagulant was acid citrate dextrose. Red cells were stored in AS-5 (Optisol®). After priming, the blood was warmed and infused at 100 mL/min via the RIS through the circuit and then through a microaggregate filter into an empty container. Blood samples were collected at two time points: (1) from the initial packet prior to filling the RIS reservoir and (2) after passing through the RIS and the microaggregate filter. Infusate temperature and peak infusion pressure were monitored and recorded. The microaggregate filter was visually inspected for evidence of a clot. All samples underwent a platelet count, which was collected in an ethylenediaminetetraacetic acid (EDTA) tube. The blood samples were collected in sodium heparin tubes and placed in a thromboelastography (TEG) cartridge (TEG 6s; Haemonetics Corporation, Braintree, MA). All blood samples were delivered to the main laboratory in a separate building by courier to undergo analysis. For data analysis, the pre-infusion samples were used as a control and compared to the post-infusion samples.

Statistical analysis was performed using JMP Pro 15.0 (SAS Institute Inc., Cary, NC). Data visualization/graphical approaches were used to assess distributional properties of data prior to analysis; since normality assumption could not be reasonably assumed, non-parametric analyses were conducted [9]. Wilcoxon signed-rank tests and non-parametric tests for paired data [10] were used for pre- to post-differences [reported as mean differences with 95% confidence intervals (CIs)] in platelet count, mean platelet volume (MPV), maximum amplitude on the TEG (MA TEG), maximum amplitude on the rapid TEG (MA rTEG), the contribution of platelets to clot strength, and maximum amplitude of the ADP on platelet mapping (MA ADP). Platelet contribution to clot strength was calculated by taking the MA TEG and subtracting the citrated blood sample activated by the functional fibrinogen test from the TEG 6s. The above values were chosen because of their relationship to platelet function and the study’s hypothesis. Spearman's correlations were used to examine relationships with the age of platelets and changes in outcomes. A p-value <0.05 was considered statistically significant. Assuming 80% power, alpha = 0.05, and correlation = 0.98 between pre- and post-values, n = 10 samples would be able to detect paired mean differences of 34 (×10^3^/µL) in platelet count [standard deviation (SD) = 50], 0.6 fL in MPV (SD = 0.9), 22 mm in clot strength (SD = 18), 5 mm in aggregation response (SD = 8), and 5 mm in MA rTEG (SD = 8).

## Results

**Figure 1:**
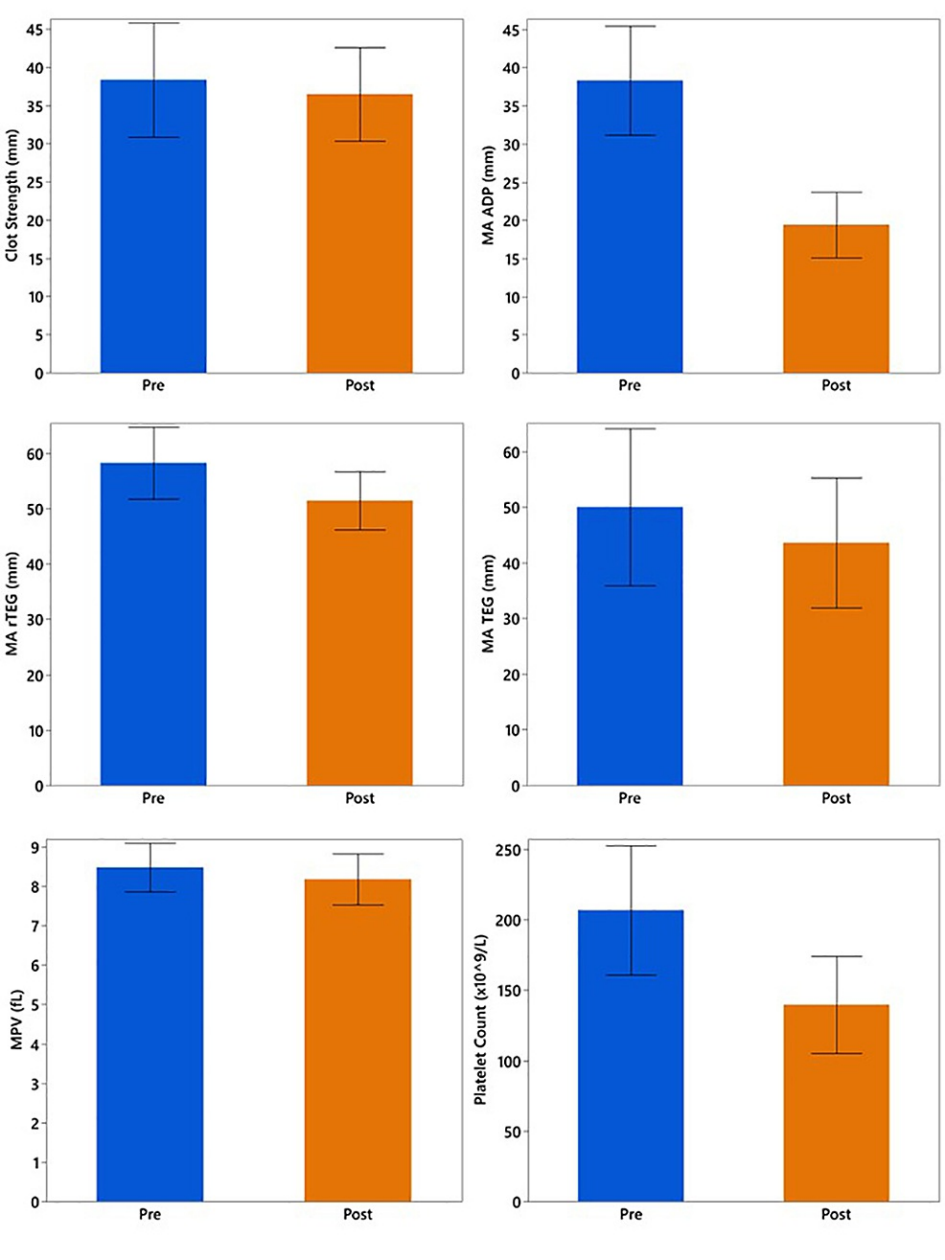
Pre- vs. post-transfusion platelet function testing for whole blood Means are reported with upper and lower 95% confidence intervals MA ADP: maximum amplitude of the adenosine diphosphate pathway; MA rTEG: maximum amplitude on rapid thromboelastography; MA TEG: maximum amplitude on the thromboelastography; MPV: mean platelet volume

The study sample (n = 10) included platelets with an average age of 5.3 days, as shown in Table 1. Prior to the study intervention, the mean clot strength was 38.3 with a mean maximum amplitude of 50.0 mm on TEG. The mean MPV and count were 8.5 fL and 206.8 × 10^3^/µL, respectively. The infusion through the RIS had a detrimental effect on all the maximal amplitudes of viscoelastic testing (Figure 1). When whole blood was infused through the RIS at a rate of 100 mL/min, it resulted in a significant reduction in MA ADP (mean difference = −18.7 mm; 95% CI: −24.1 to −13.3, P = 0.004), MA rTEG (mean difference = −6.0; 95% CI: −10.0 to −2.0, P = 0.008), MA TEG (mean difference = −7.1; 95% CI: −10.9 to −3.4, P = 0.004). Decreases were also seen in MPV (mean difference = −0.3; 95% CI: −0.6 to −0.1, P = 0.02), and platelet count (mean difference = −68.3 × 10^3^/µL; 95% CI: −86.9 to −49.7, P = 0.004, Figure 1). There was no reduction in viscoelastic strength from platelet contribution (mean difference = −1.8; 95% CI: −8.3 to 4.5, P = 0.57). Additionally, no visible clot formation was observed on any microaggregate filters, and infusing pressure never exceeded 76 mmHg.

**Table 1:** Blood characteristics and baseline (pre-transfusion) measurements SD: standard deviation; MA ADP: maximum amplitude of the adenosine diphosphate pathway; MA rTEG: maximum amplitude on rapid thromboelastography; MA TEG: maximum amplitude on the thromboelastography; MPV: mean platelet volume

Variables	Whole blood (n = 10)
Age of platelet, days (mean ± SD)	5.3 ± 2.2
Clot strength, mm (mean ± SD)	38.3 ± 9.7
MA ADP, mm (mean ± SD)	38.3 ± 10.0
MA rTEG, mm (mean ± SD)	58.2 ± 9.1
MA TEG, mm (mean ± SD)	50.0 ± 19.7
MPV, fL (mean ± SD)	8.5 ± 0.9
Platelet count (×10^3^/µL) (mean ± SD)	206.8 ± 64.2

Table 2 reports correlations of the age of platelets with change in outcomes pre- to post-transfusion. While moderate correlations were observed between age of platelets and change in MA ADP (rho = 0.43, P = 0.22) and change in MA TEG (rho = 0.49, P = 0.15), these associations did not reach statistical significance in our sample. Likewise, the age of platelets was not statistically significantly correlated with change in any other outcome (Table 2).

**Table 2:** Spearman correlation between age of platelets and change in pre- to post-transfusion values MA ADP: maximum amplitude of the adenosine diphosphate pathway; MA rTEG: maximum amplitude on rapid thromboelastography; MA TEG: maximum amplitude on the thromboelastography; MPV: mean platelet volume

Variables	Correlation (Spearman’s rho) with the age of platelets	P-value
Pre- to post-transfusion change in clot strength	0.02	0.96
Pre- to post-transfusion change in MA ADP	0.43	0.22
Pre- to post-transfusion change in MA rTEG	–0.17	0.64
Pre- to post-transfusion change in MA TEG	0.49	0.15
Pre- to post-transfusion change in MPV	–0.25	0.48
Pre- to post-transfusion change in platelet count (×10^3^/µL)	0.09	0.80

## Discussion

The results of our study suggest that the rapid infusion of whole blood via the Belmont Fluid Management System is associated with negative effects on platelets. Because of the reduction in platelet volume and platelet number, it is likely that either platelet destruction and/or aggregation played a role in the negative effects. The effects on platelets did not cause a decrease in clot strength. While negative effects on platelets were observed in this study, it is doubtful that these effects are clinically significant due to their small size. In a scenario where a RIS is involved, it could transfuse platelets much faster and in an equal ratio to other blood components.

Notably, Konig et al. found that there was no significant decrease in platelet function as measured by aggregometry observed in warmed samples versus control [11]. A likely reason that our results differed from Konig et al. is that our study processed fluid through a RIS, which not only warms blood but also rapidly infuses it with a roller pump mechanism. The study by Konig et al. suggested that warming by itself does not cause the results we observed. The use of an electromechanical infusion pump also does not seem to confer damage to platelets [12]. Rather, our findings may result from either shear stress from high flows through a roller pump, similar to what is observed during centrifugation, or platelet activation causing agglutination inside the tubing of the RIS. A reduction in aggregation response has been seen with as little as 100 dynes/cm^2^ shear stress, and for as short a time as 30 seconds [13].

There is a growing body of literature published surrounding infusing platelets through a RIS since this study was completed. Zaza et al. performed 10 iterations of infusing whole blood through a Belmont FMS 2000, and samples were evaluated using platelet counts, multiplate, TEG, and a calibrated automated thrombogram (Abstract: Zaza M, Wang Y, George M, Wade CE, Cardenas JC, Cotton BA. Impact of Rapid Transfusion Devices on Whole Blood Platelet Count, Platelet Function, & Hemostatic Potential. Military Health System Research Symposium; 2018). They found a significant reduction in platelet count at 70 mL/min and 100 mL/min and differences in thrombin generation, but no reduction in the MA on TEG. Hess et al. [14] performed 10 iterations of infusing platelets stored in plasma through a Belmont RI-2 and samples were evaluated using platelet count and TEG. They did not find any significant differences between pre-infusate and post-infusate. Alley et al. [15] performed 10 iterations of infusing whole blood through a Smiths Medical Level 1 H-1000 Fast Flow Fluid Warmer (Smiths Medical, Inc., Minneapolis, MN) and samples were evaluated using rTEG and platelet aggregation. Alley et al. also did not find any significant differences. In all three of these studies, no microaggregate filter was placed at the end, which may have contributed to some of the differences seen between this study and the previously published studies.

The finding of a lower MA ADP is not a completely benign finding in the literature. For patients arriving in the trauma bay, even a reduction of 16% of the ADP using multiple electrode aggregometry is associated with increased mortality [16]. Reduced platelet aggregation in response to ADP has been associated with increased transfusion requirements in trauma patients [17]. However, the effect of transfusing platelets with an already reduced MA ADP has not been previously described. The literature does not describe whether a reduction in MA ADP is clinically relevant when platelets are administered at a faster rate associated with the need for a RIS. Shear stress may contribute to the decreased platelet viscoelastic clot strength and platelet aggregation response to ADP activation observed in this study. Centrifugation of platelets has been shown to cause the release of β-thromboglobulin and lactic dehydrogenase [18], neither of which we examined. Some of the platelet storage lesion from apheresis is reversible [19]. It is possible that some of the platelet dysfunction secondary to transfusion through a RIS seen in this study is also reversible.

Recent data from wars in Iraq and Afghanistan suggest that whole blood transfusion in the early resuscitation phase may decrease mortality and morbidity [20]. As a consequence, the use of whole blood for massive transfusion of blood products in civilian hemorrhagic shock is increasing. Data from studies of cardiopulmonary bypass and liver transplants suggest that fresh whole blood transfusions yield greater platelet aggregation on extracellular matrices and less dilutional coagulopathy compared with platelet transfusion alone [21,22]. This is usually not a problem when using component blood therapy for massive transfusions because platelets are generally administered via a separate line, usually infusing at a slower rate. The option to infuse platelets in a non-warmed line separate and apart from the PRBC and fresh frozen plasma components is common practice.

This study has several limitations. The biggest limitation was the lack of a pressure bag infusion control group. It is unknown how much greater the effect of platelet loss and decrease in viscoelastic strength of the tested samples that were infused through the heating element and roller pump in the Belmont would be compared to running blood samples infused via pressure bag and having the tubing heated to 37 °C. It is also unclear if reheating to 37 °C a second time during analysis caused additional or more damage to the platelets than just the warming through the RIS. While our study shows compelling evidence of decreased platelet count and viscoelastic strength after whole blood transfusion via a RIS, it is difficult to assess the relative risk compared to current practice. A second limitation is that platelet loss or reduction in platelet size was assumed to be from platelet lysis. Platelets may have become activated and stuck to the tubing, microaggregate filter, or containers, particularly around the roller pump mechanism via agglutination. This would not be observed in the microaggregate filter. Another potential limitation is that expired platelets were used. We believe this was justified because platelets are stored at room temperature and, therefore, platelet expiration is related to the risk of infection rather than alterations in platelet function [23]. AuBuchon et al. reported that platelets transfused seven days after the collection had 84.5% of the total amount and 75.9% of the survival of platelets transfused two to four hours after collection [24]. They also demonstrated that five-day-old platelets had 77.9% recovery and 91.8% survival compared with platelets transfused within one day of collection [25].

Another limitation in the analysis is that the TEG and platelet mapping analyses used in this study are not the gold standard for platelet function testing like platelet aggregometry. Viscoelastic testing, including platelet mapping and rapid TEG, has been used in other studies as an acceptable surrogate, as well as in clinical protocols for the administration of platelets [17,26,27]. Despite the less sensitive tests we used, we still identified a reduction in viscoelastic clot strength and platelet aggregation response to ADP activation. Thus, we assume that a more sensitive test would also have confirmed our findings and similarly rejected the null hypothesis. Two areas that remain unstudied are whether transfusing warmed platelets either through a RIS or not activates platelets or causes microaggregates.

## Conclusions

An infusion of whole blood through a RIS is possibly associated with decreased platelet quantity and a reduction in viscoelastic strength, at least in one device. Further study is required to determine if this is an artifact from our study design and if the observed effects are reversible. Additionally, it remains to be decided whether these differences are clinically relevant.
